# Insecticide Mixtures Could Enhance the Toxicity of Insecticides in a Resistant Dairy Population of *Musca domestica* L

**DOI:** 10.1371/journal.pone.0060929

**Published:** 2013-04-16

**Authors:** Hafiz Azhar Ali Khan, Waseem Akram, Sarfraz Ali Shad, Jong-Jin Lee

**Affiliations:** 1 Department of Entomology, Bahauddin Zakariya University, Multan, Pakistan; 2 Department of Agri. Entomology, University of Agriculture, Faisalabad, Pakistan; 3 Chonbuk National University, Chonju, South Korea; Universidade Federal do Rio de Janeiro, Brazil

## Abstract

House flies, *Musca domestica* L., are important pests of dairy operations worldwide, with the ability to adapt wide range of environmental conditions. There are a number of insecticides used for their management, but development of resistance is a serious problem. Insecticide mixtures could enhance the toxicity of insecticides in resistant insect pests, thus resulting as a potential resistance management tool. The toxicity of bifenthrin, cypermethrin, deltamethrin, chlorpyrifos, profenofos, emamectin benzoate and fipronil were assessed separately, and in mixtures against house flies. A field-collected population was significantly resistant to all the insecticides under investigation when compared with a laboratory susceptible strain. Most of the insecticide mixtures like one pyrethroid with other compounds evaluated under two conditions (1∶1-“A” and LC_50_: LC_50_-“B”) significantly increased the toxicity of pyrethroids in the field population. Under both conditions, the combination indices of pyrethroids with other compounds, in most of the cases, were significantly below 1, suggesting synergism. The enzyme inhibitors, PBO and DEF, when used in combination with insecticides against the resistant population, toxicities of bifenthrin, cypermethrin, deltamethrin and emamectin were significantly increased, suggesting esterase and monooxygenase based resistance mechanism. The toxicities of bifenthrin, cypermethrin and deltamethrin in the resistant population of house flies could be enhanced by the combination with chlorpyrifos, profenofos, emamectin and fipronil. The findings of the present study might have practical significance for resistance management in house flies.

## Introduction

The house fly, *Musca domestica* L., has been considered as one of the major pests of dairies and public health with a potential role in disease transmission, nuisance to animals and humans, and have the ability to disperse off-farm areas [1]. Various insecticides from organochlorine, organophosphate, carbamate, pyrethroid and new chemistry classes have been used for their management worldwide. However, they have the ability to develop resistance to most of the insecticides used for their management all over the world.

Insecticide mixtures, rotation and/or fine scale mosaics have been proposed as important tools for resistance management in different insect pests [2]. Mixtures consisted of organophosphate, pyrethroid or carbamate insecticides have been found very effective in enhancing the toxicity of insecticides in different resistant insect pests worldwide like *Bemisia tabaci* [3], *Culex quinquefasciatus* [4], *Helicoverpa armigera* [5], [6], *Musca domestica* [7] and *Spodoptera litura* [8]. This type of potentiation or synergism is explained by the inhibition of esterases [9], [10] or monooxygenases activity [6]. However, cases of insecticide resistance to different classes of insecticides are increasing day by day. This situation is alarming, as there are very few classes, and the development of new insecticides is limited owing to the rising standards for environmental safety [11]. In this, alternative strategies like mosaics or rotational use of insecticides with different modes of action should also be included in resistance management programs [12].

Theoretically, mixing insecticides (with different modes of action) usually prove very effective in resistance management programs compared to mosaics or rotational use of insecticides [13] because, if a resistance mechanism to each insecticide in the mixture is independent and initially rare, the chances for the occurrence of resistance to both insecticides at the same time would be minimum [14]. There are a number of studies on mixture toxicities (particularly pyrethroids with other compounds) in different dipteran insect pests worldwide, however, to the best of authors’ knowledge such studies are rare in the house fly research particularly in Pakistan. Since pyrethroids and organophosphates have different modes of action, their mixtures have commonly been in practice against a variety of pests worldwide for the last many years [8]. Increased metabolic detoxification is one of the major mechanisms in house flies for the development of insecticide resistance [15]. Previously it has been assumed that organophosphates, when used in combination with pyrethroids, inhibit the enzymes responsible for metabolic detoxification in different insect pests [6], [9], [10]. Given the development of insecticide resistance in field populations of house flies from the dairies in Punjab, Pakistan [16], [17], an investigation was made using different combination of pyrethroids (bifenthrin, cypermethrin, deltamethrin) with organophosphates (chlorpyrifos, profenofos) and new insecticides (emamectin benzoate, an avermectin; and fipronil, a phenypyrazole), to study the synergistic or antagonistic interaction between insecticides used in mixtures against house flies. The paper also addresses the question of synergistic interaction of two synergists, piperonyl butoxide (PBO) and S,S,S-tributylphosphorotrithioate (DEF), with different insecticides under investigation.

## Methods

### Insects

The adult flies were collected from a dairy farm of the province Punjab, Pakistan. Recently, resistance to different insecticides have been observed in house flies from the said area [16], [17]. No specific permit was required to collect house fly samples from the dairy farm as it was privately owned and collection was made merely by speaking with the private owner. Since the house fly is not an endangered species, no such permission was required from any concerned authority in Punjab, Pakistan. The collected flies were fed on powdered milk, sugar and water while larvae were reared on a medium of milk powder, sugar, yeast, grass meal and wheat bran with a ratio of 0.3∶0.3∶1∶2∶4 by weight, respectively [18]. A population of house flies was collected from zero or very low chemical use zone and maintained in the laboratory without any chemical exposure. This population was designated as the laboratory susceptible strain. The fly cultures were maintained in the laboratory at 25±2°C, 60±5% RH and 12L: 12D (h) photoperiod.

### Chemicals

Commercial formulations of the following insecticides were used for bioassays: bifenthrin (Talstar ® 100 EC, FMC) cypermethrin (Arrivo® 10 EC, FMC), deltamethrin (Decis Super®, 10.5 EC, Bayer Crop Science), profenofos (Curacron® 50 EC, Syngenta), chlorpyrifos, emamectin benzoate (Proclaim® 019EC, Syngenta) and fipronil (Regent® 36EC, Bayer Crop Sciences). Two synergists, *S*,*S*,*S*-tributylphosphorotrithioate (DEF; Sigma Ltd, UK), the esterase specific inhibitor, and piperonyl butoxide (PBO; Sigma Ltd, UK), an inhibitor of cytochrome P450 monooxygenases and of esterases were also used to check their effect on insecticide toxicity.

### Bioassays

Feeding bioassay method was used to assess the toxicities of insecticides under investigation [19]. Briefly, twenty 3–5 days old female flies were introduced in plastic containers (250 ml) and provided two pieces of cotton dental wick (2 cm length) moistened with 20% sugar water solution containing an insecticide or an insecticide mixture concentration. Five to eight concentrations were prepared for each insecticide and each concentration was replicated three times. In control plastic jars, cotton wicks soaked in 20% sugar solution without toxicant were given to flies. To avoid drying, cotton wicks were hydrated at 24 hours [19]. Bioassays were conducted at 25±2°C, 60±5% RH and 12∶12 (L/D) photoperiod. Synergists PBO and DEF at the maximum sublethal dose of 10 µg per fly (in 0.5 µl acetone) were applied 1 h before the insecticide treatment as reported elsewhere [20]. Final mortality was assessed after 48 h of exposure to insecticides and the flies were assumed dead if they were ataxic. LC_50s_ were calculated by Probit Analysis using the software SPSS 10 for windows.

### Synergism analysis

The combination index (CI) method proposed by Chou and Talalay [21], was used to evaluate possible additive, antagonistic or synergistic interaction between the insecticides in the mixture. To determine which of the above said interaction exist between the insecticides in the mixture, two insecticides in the mixtures were tested under two conditions viz., 1∶1-“A” and LC_50_: LC_50_-“B”, with serial concentrations. **To assess synergism, antagonism and/or additive effect**, the combination index was determined by using the following equation:
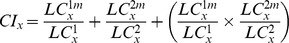
Where the values in the numerator of the equation are the lethal concentrations of insecticide 1 and 2 respectively, giving the mortality x, while the values in the denominator are the lethal concentrations of insecticide 1 and 2 respectively, producing the same mortality x when used alone. The resultant values of CIs were scaled to categorize the mixture effect: additive effect when combination index  = 1, antagonistic effect when combination index >1, and synergistic effect when combination index <1 [21]. These values were calculated at 50% mortality level.

## Results

### Toxicity of insecticides alone or in mixture to the laboratory susceptible strain

The relative toxicities of organophosphates and new chemical insecticides were significantly higher (non overlapping of 95% CL; P<0.01) than that of the pyrethroids (Table 1).

**Table 1:** Toxicity of insecticides alone and in combination against laboratory susceptible strain of *Musca domestica.*

Insecticide	Ratio	n*	LC_50_ (95% CL)**	Slope ± SE	χ^2^	df	P
Bifenthrin	1:0	360	10.89 (9.18–13.11)	2.23±0.23	2.52	3	0.47
Cypermethrin	1:0	360	4.90 (4.24–5.59)	2.86±0.27	2.56	3	0.47
Deltamethrin	1:0	420	13.18 (11.18–15.59)	2.25±0.19	1.66	4	0.79
Chlorpyrifos	1:0	420	1.85 (1.55–2.18)	2.28±0.20	0.49	4	0.98
Profenofos	1:0	420	1.81 (1.53–2.14)	2.23±0.19	1.55	4	0.82
Emamectin benzoate	1:0	360	2.89 (2.46–3.46)	2.34±0.24	1.44	3	0.69
Fipronil	1:0	360	1.94 (1.62–2.40)	2.19±0.24	1.83	3	0.61
Bifenthrin + Chlorpyrifos	1:1	420	6.69 (5.29–8.90)	1.53±0.17	0.86	4	0.93
Bifenthrin + Profenofos	1:1	420	5.32 (4.20–7.06)	1.47±0.16	0.64	4	0.96
Bifenthrin + Emamectin	1:1	420	4.52 (3.75–5.51)	1.90±0.18	6.64	4	0.17
Bifenthrin + Fipronil	1:1	420	2.96 (2.39–3.69)	1.57±0.16	1.79	4	0.78
Cypermethrin + Chlorpyrifos	1:1	420	3.33 (2.71–3.55)	1.81±0.18	6.71	4	0.15
Cypermethrin + Profenofos	1:1	360	7.10 (5.51–10.12)	1.71±0.23	0.61	3	0.90
Cypermethrin + Emamectin	1:1	420	2.51 (2.02–3.20)	1.59±0.16	0.96	4	0.92
Cypermethrin + Fipronil	1:1	420	4.21 (3.98–5.44)	1.71±0.18	3.29	4	0.51
Deltamethrin + Chlorpyrifos	1:1	420	8.77 (7.25–10.97)	2.00±0.18	3.08	4	0.55
Deltamethrin + Profenofos	1:1	420	8.82 (6.03–15.11)	1.93±0.19	7.81	4	0.10
Deltamethrin + Emamectin	1:1	480	4.90 (4.12–5.89)	2.04±0.16	6.79	5	0.24
Deltamethrin + Fipronil	1:1	360	9.88 (7.83–13.18)	1.63±0.17	0.83	3	0.94
Chlorpyrifos + Bifenthrin	1:5.89	360	3.45 (2.76–4.32)	1.63±0.19	0.86	3	0.83
Profenofos + Bifenthrin	1:6.02	360	3.44 (2.86–4.15)	2.04±0.22	2.41	3	0.49
Emamectin + Bifenthrin	1:3.77	420	2.65 (2.18–3.23)	1.79±0.16	3.09	4	0.54
Fipronil + Bifenthrin	1:5.61	420	1.38 (1.33–1.95)	1.91±0.18	1.47	4	0.83
Chlorpyrifos + Cypermethrin	1:2.65	360	2.28 (1.91–2.79)	2.03±0.22	4.97	3	0.17
Profenofos + Cypermethrin	1:2.71	360	3.22 (2.23–5.40)	2.34±0.35	5.34	3	0.15
Emamectin + Cypermethrin	1:1.70	360	1.24 (0.83–1.90)	2.36±0.23	6.28	3	0.10
Fipronil + Cypermethrin	1:2.53	360	1.55 (1.30–1.84)	2.22±0.23	3.77	3	0.29
Chlorpyrifos + Deltamethrin	1:7.12	360	5.22 (4.35–6.36)	2.08±0.22	3.17	3	0.37
Profenofos + Deltamethrin	1:7.28	360	5.80 (4.85–7.11)	2.11±0.22	1.83	3	0.61
Emamectin + Deltamethrin	1:4.56	360	3.46 (2.92–4.07)	2.35±0.24	3.82	3	0.28
Fipronil + Deltamethrin	1:6.79	420	2.71 (2.30–3.20)	2.25±0.19	2.37	4	0.67

* number of females exposed.

** lethal conc. (µg/ml) to kill fifty percent population.

The toxicities of pyrethroids, based on their LC_50s_, increased when mixed with other insecticides. For bifenthrin, this effect was observed in all combinations: bifenthrin/other non-pyrethroids. The toxicity of bifenthrin increased significantly when mixed with fipronil over that of mixtures with the other non-pyrethroids at the both ratios tested. For cypermethrin, the increased toxicity was observed in the following combinations: cypermethrin/chlorpyrifos or emamectin at the 1∶1 ratio, and cypermethrin/other insecticides except profenofos, at the LC_50_: LC_50_ ratio. At the 1∶1 ratio, the mixtures of cypermethrin/chlorpyrifos or emamectin were significantly more toxic compared to the mixture with profenofos or fipronil while at LC_50_: LC_50_ ratio, the mixture of cypermethrin with either emamectin or fipronil was significantly more toxic than the rest of the two mixtures. For deltamethrin, the mixture of deltamethrin with either chlorpyrifos or emamectin at the 1∶1 ratio, and with all the other insecticides at the LC_50_: LC_50_ ratio, showed significantly increased toxicities compared to the toxicity of deltamethrin used alone. At the 1∶1 ratio, the toxicity of deltamethrin/emamectin was higher than the rest of the three mixtures while at the LC_50_: LC_50_ ratio, the mixtures of deltamethrin with either emamectin or fipronil were significantly more toxic than the rest of the two mixtures. Moreover, the toxicities of pyrethroids/non-pyrethroids were significantly higher at the LC_50_: LC_50_ ratio (non overlapping 95% CL; P<0.01) than the combinations mixed at the 1∶1 ratio, except the toxicities of following combinations: cypermethrin/chlorpyrifos and deltamethrin/profenofos (Table 1).

The mixtures of bifenthrin, except with fipronil revealed antagonistic effect at the 1∶1 ratio with combination index values >1, however, at the LC_50_: LC_50_ ratio all combinations produced synergistic effect with combination index values <1 (Table 2). For cypermethrin at the 1∶1 ratio, only cypermethrin/emamectin mixture produced a synergistic effect while at the LC_50_: LC_50_ ratio, except cypermethrin/profenofos, all the mixtures produced a synergistic effect. Similarly, the mixtures of deltamethrin/other insecticides at the 1∶1 ratio revealed antagonistic effect, while snyrgistic effect was observed at the other ratio tested (Table 2).

**Table 2:** Combination index (CI) values of insecticide mixtures against the laboratory susceptible strain of *Musca domestica.*

Insecticide mixture (A+B)	Active ingredient ratio			At LC_50_ level
	Ratio	A*	B*	CI**
Bifenthrin + Chlorpyrifos	1:1	3.35	3.35	1.20
Bifenthrin + Profenofos	1:1	2.66	2.66	2.07
Bifenthrin + Emamectin	1:1	2.26	2.26	1.15
Bifenthrin + Fipronil	1:1	1.48	1.48	1.00
Cypermethrin + Chlorpyrifos	1:1	1.66	1.66	1.54
Cypermethrin + Profenofos	1:1	3.55	3.55	4.10
Cypermethrin + Emamectin	1:1	1.26	1.26	0.80
Cypermethrin + Fipronil	1:1	2.11	2.11	1.99
Deltamethrin + Chlorpyrifos	1:1	4.38	4.38	2.88
Deltamethrin + Profenofos	1:1	4.41	4.41	2.95
Deltamethrin + Emamectin	1:1	2.45	2.45	1.191.19
Deltamethrin + Fipronil	1:1	4.94	4.94	3.88
Chlorpyrifos + Bifenthrin	1:5.89	0.50	2.95	0.62
Profenofos + Bifenthrin	1:6.02	0.49	2.95	0.62
Emamectin + Bifenthrin	1:3.77	0.55	2.09	0.42
Fipronil + Bifenthrin	1:5.61	0.21	1.71	0.23
Chlorpyrifos + Cypermethrin	1:2.65	0.62	1.66	0.80
Profenofos + Cypermethrin	1:2.71	0.87	2.35	1.19
Emamectin + Cypermethrin	1:1.70	0.46	0.78	0.35
Fipronil + Cypermethrin	1:2.53	0.44	1.11	0.50
Chlorpyrifos + Deltamethrin	1:7.12	0.64	4.58	0.81
Profenofos + Deltamethrin	1:7.28	0.70	5.10	0.91
Emamectin + Deltamethrin	1:4.56	0.62	2.84	0.49
Fipronil + Deltamethrin	1:6.79	0.35	2.36	0.39

* calculated value of insecticide A and B (µg/ml) respectively.

** combination index.

### Toxicity of insecticides alone or in mixture to the field collected strain

The field collected population showed resistance to all the tested insecticides when compared with the laboratory reared susceptible strain (Table 3). The resistance ratios were 11.72, 64.79, 15.87, 8.82, 22.02, 43.11 and 22.29 for bifenthrin, cypermethrin, deltamethrin, chlorpyrifos, profenofos, emamectin and fipronil, respectively, when these insecticides used alone.

**Table 3:** Toxicity of insecticides alone and in combination against field strain of *Musca domestica.*

Insecticide	Ratio	n*	LC_50_ (95% CL)**	Slope ± SE	χ^2^	df	P	RR***
Bifenthrin	1:0	420	127.62 (104.75–157.69)	1.74±0.17	1.74	4	0.78	11.72
Cypermethrin	1:0	480	317.45 (250.39–418.56)	1.39±0.14	1.73	5	0.88	64.79
Deltamethrin	1:0	540	209.12 (170.00–262.11)	1.55±0.12	7.93	6	0.24	15.87
Chlorpyrifos	1:0	360	16.31 (13.17–21.29)	1.81±0.22	0.02	3	0.99	8.82
Profenofos	1:0	480	39.85 (32.89–49.41)	1.84±0.16	7.74	5	0.17	22.02
Emamectin benzoate	1:0	420	124.59 (97.82–168.87)	1.54±0.17	0.38	4	0.98	43.11
Fipronil	1:0	420	43.24 (30.34–67.40)	2.01±0.19	7.56	4	0.11	22.29
Bifenthrin + Chlorpyrifos	1:1	420	44.64 (37.03–55.00)	1.94±0.18	4.20	4	0.38	6.67
Bifenthrin + Profenofos	1:1	420	58.39 (46.23–77.22)	1.60±0.16	1.23	4	0.87	10.98
Bifenthrin + Emamectin	1:1	360	141.00 (113.02–187.47)	1.81±0.22	1.48	3	0.69	31.19
Bifenthrin + Fipronil	1:1	360	100.36 (80.04–134.71)	1.81±0.22	3.74	3	0.29	33.91
Cypermethrin + Chlorpyrifos	1:1	360	74.09 (60.73–90.64)	1.86±0.21	0.15	3	0.98	22.32
Cypermethrin + Profenofos	1:1	420	51.87 (41.94–64.17)	1.60±0.16	1.68	4	0.80	7.31
Cypermethrin + Emamectin	1:1	480	77.45 (64.50–94.45)	1.91±0.16	7.27	5	0.20	30.86
Cypermethrin + Fipronil	1:1	420	64.74 (50.43–85.00)	1.29±0.15	1.50	4	0.82	15.38
Deltamethrin + Chlorpyrifos	1:1	480	93.01 (76.81–115.31)	1.88±0.17	5.75	5	0.33	10.64
Deltamethrin + Profenofos	1:1	420	152.72 (116.77–216.41)	1.39±0.12	1.16	4	0.89	17.32
Deltamethrin + Emamectin	1:1	480	90.67 (73.35–115.36)	1.60±0.15	3.30	5	0.65	18.50
Deltamethrin + Fipronil	1:1	480	69.54 (54.96–90.73)	1.35±0.13	0.51	5	0.99	7.04
Chlorpyrifos + Bifenthrin	1:7.82	420	29.33 (24.16–35.85)	1.78±0.17	4.25	4	0.37	8.50
Profenofos + Bifenthrin	1:3.20	420	26.57 (17.10–40.91)	1.67±0.16	7.89	4	0.10	7.72
Emamectin + Bifenthrin	1:1.02	480	60.51 (48.78–76.74)	1.50±0.14	1.02	5	0.96	22.92
Fipronil + Bifenthrin	1:2.95	420	96.82 (73.54–139.51)	1.40±0.17	1.10	4	0.89	70.16
Chlorpyrifos + Cypermethrin	1:19.46	420	54.79 (45.09–66.50)	1.80±0.17	2.25	4	0.69	24.03
Profenofos + Cypermethrin	1:7.97	420	45.85 (35.96–60.26)	1.35±0.15	0.34	4	0.99	14.23
Emamectin + Cypermethrin	1:2.55	420	93.46 (64.89–138.78)	1.97±0.18	7.76	4	0.10	75.37
Fipronil + Cypermethrin	1:7.34	480	90.39 (75.49–109.67)	1.94±0.16	1.73	5	0.89	58.32
Chlorpyrifos + Deltamethrin	1:12.82	420	45.59 (37.15–56.36)	1.66±0.16	2.14	4	0.71	8.73
Profenofos + Deltamethrin	1:5.25	360	53.05 (32.93–82.71)	2.01±0.23	5.56	3	0.14	9.51
Emamectin + Deltamethrin	1:1.68	480	102.84 (79.24–140.15)	1.24±0.12	0.78	5	0.98	29.72
Fipronil + Deltamethrin	1:4.84	420	44.08 (36.03–53.91)	1.72±0.16	3.08	4	0.55	16.24

* number of females exposed.

** lethal conc. (µg/ml) to kill the fifty percent population.

*** resistance factor, calculated as [LC_50_ of field population ÷ LC_50_ of laboratory susceptible strain].

In most of the cases, the toxicities of pyrethroids, based on their LC_50s_, increased significantly when mixed with other insecticides. For bifinthrin, the toxicity was increased significantly with the following mixtures: bifenthrin/organophosphates at the 1∶1 ratio, and bifenthrin/other insecticides except fipronil at the LC_50_: LC_50_ ratio. However, bifenthrin with either emamectin or fipronil at 1∶1, and bifenthrin/fipronil at LC_50_: LC_50_ ratio did not increase toxicity since their LC_50_ values were similar with bifenthrin alone based on overlapping of 95% CLs. In case of cypermethrin, either it was used at the ratio of 1∶1 or LC_50_: LC_50_ with the other insecticides, the toxicities increased significantly compared to the toxicity of cypermethrin alone. Similarly, the toxicity of deltamethrin increased significantly with all the combinations at both tested ratios except the combination with profenofos at the 1∶1 ratio where the LC_50_ value was the same as for deltamethrin alone (Table 3).

For each mixture, the combination index was calculated for the purpose to assess possible additive, antagonistic or synergistic interaction between insecticides. The mixtures of bifenthrin/other insecticides produced an antagonistic effect (combination index >1) at the 1∶1 ratio, however, synergistic effect was observed (combination index <1) with all the combinations when they were tested in the ratio of LC_50_: LC_50_, except bifenthrin/fipronil mixture (combination index >1) (Table 4). For cypermethrin, all the tested combinations produced a synergistic effect in both ratios tested, except the combination cypermethrin/chlorpyrifos which was antagonistic when used at the 1∶1 ratio. In case of deltamethrin at the 1∶1 ratio, only the combination deltamethrin/emamectin produced synergistic effect. In contrast, all the combinations of deltamethrin with other insecticides gave synergistic effect when used in the ratio of LC_50_: LC_50_ (Table 4).

**Table 4:** Combination index (CI) values of insecticide mixtures against field strain of *Musca domestica.*

Insecticide mixture (A+B)	Active ingredient ratio			At LC_50_ level
	Ratio	A*	B*	CI**
Bifenthrin + Chlorpyrifos	1:1	22.32	22.32	1.79
Bifenthrin + Profenofos	1:1	29.20	29.20	1.13
Bifenthrin + Emamectin	1:1	70.50	70.50	1.42
Bifenthrin + Fipronil	1:1	50.18	50.18	2.01
Cypermethrin + Chlorpyrifos	1:1	37.05	37.05	2.66
Cypermethrin + Profenofos	1:1	25.94	25.94	0.78
Cypermethrin + Emamectin	1:1	38.73	38.73	0.47
Cypermethrin + Fipronil	1:1	32.37	32.37	0.93
Deltamethrin + Chlorpyrifos	1:1	46.51	46.51	3.71
Deltamethrin + Profenofos	1:1	76.36	76.36	3.00
Deltamethrin + Emamectin	1:1	45.34	45.34	0.66
Deltamethrin + Fipronil	1:1	34.77	34.77	1.11
Chlorpyrifos + Bifenthrin	1:7.82	3.33	26.00	0.45
Profenofos + Bifenthrin	1:3.20	6.33	20.24	0.34
Emamectin + Bifenthrin	1:1.02	29.96	30.55	0.54
Fipronil + Bifenthrin	1:2.95	24.51	72.31	1.46
Chlorpyrifos + Cypermethrin	1:19.46	2.68	52.11	0.36
Profenofos + Cypermethrin	1:7.97	5.11	40.74	0.27
Emamectin + Cypermethrin	1:2.55	26.33	67.13	0.47
Fipronil + Cypermethrin	1:7.34	10.84	79.55	0.57
Chlorpyrifos + Deltamethrin	1:12.82	3.30	42.29	0.44
Profenofos + Deltamethrin	1:5.25	8.49	44.56	0.40
Emamectin + Deltamethrin	1:1.68	30.37	72.47	0.72
Fipronil + Deltamethrin	1:4.84	7.55	36.53	0.38

* calculated value of insecticide A and B (µg/ml), respectively.

** combination index.

### Effect of enzyme inhibitors on the toxicities of insecticides

The use of two enzyme inhibitors viz., PBO and DEF, against the field population of *M. domestica* largely overcame resistance to bifenthrin, cypermethrin, deltamethrin, chlorpyrifos, profenofos and emamectin. PBO significantly reduced LC_50_ values (non overlapping of 95% CLs) for bifenthrin from 127.62 to 21.94 (6 fold), cypermethrin from 317.45 to 43.86 (7 fold), deltamethrin from 209.12 to 38.76 (5 fold), chlorpyrifos from 16.31 to 6.19 (3 fold), profenofos from 39.85 to 14.77 (3 fold), and emamectin from 124.59 to 60.61 (2 fold). However, PBO did not produce a synergistic effect with fipronil. DEF also reduced LC_50_ values for bifenthrin from 127.62 to 64.35 (2 fold), cypermethrin from 317.45 to 162.50 (2 fold), deltamethrin from 209.12 to 100.09 (2 fold), chlorpyrifos from 16.31 to 3.66 (4 fold), and profenofos from 39.85 to 5.48 (7 fold). However, DEF did not show synergism with emamectin and fipronil (Table 5). Moreover, PBO and DEF did not synergize any insecticide against the laboratory susceptible strain.

**Table 5:** Effect of enzyme inhibitors on insecticide toxicity against *Musca domestica.*

Strain	Insecticide	LC_50_ (95% CL)	Slope±SE	χ^2^	df	P	RR	SR*
Lab	Bifenthrin	10.89 (9.18–13.11)	2.23±0.23	2.52	3	0.47		
	Cypermethrin	4.90 (4.24–5.59)	2.86±0.27	2.56	3	0.47		
	Deltamethrin	13.18 (11.18–15.59)	2.25±0.19	1.66	4	0.79		
	Chlorpyrifos	1.85 (1.55–2.18)	2.28±0.20	0.49	4	0.98		
	Profenofos	1.81 (1.53–2.14)	2.23±0.19	1.55	4	0.82		
	Emamectin	2.89 (2.46–3.46)	2.34±0.24	1.44	3	0.69		
	Fipronil	1.94 (1.62–2.40)	2.19±0.24	1.83	3	0.61		
	Bifenthrin+PBO	8.02 (6.53–9.86)	1.80±0.21	0.86	3	0.84		1
	Bifenthrin+DEF	11.83 (8.06–19.69)	1.92±0.19	8.19	4	0.09		1
	Cypermethrin+PBO	3.84 (2.49–5.95)	2.32±0.23	6.36	3	0.10		1
	Cypermethrin+DEF	5.60 (4.74–6.70)	2.34±0.24	4.33	3	0.22		1
	Deltamethrin+PBO	9.59 (7.78–11.96)	1.73±0.20	3.72	3	0.29		1
	Deltamethrin+DEF	16.14 (13.84–18.91)	2.54±0.22	2.18	4	0.70		1
	Chlorpyrifos+PBO	2.54 (2.10–3.03)	2.23±0.23	0.30	3	0.96		1
	Chlorpyrifos+DEF	1.54 (1.29–1.83)	2.26±0.22	1.42	3	0.70		1
	Profenofos+PBO	1.97 (1.66–2.37)	2.08±0.18	2.62	4	0.62		1
	Profenofos+DEF	1.27 (1.02–1.62)	1.60±0.20	1.14	3	0.77		1
	Emamectin+PBO	3.26 (2.73–3.99)	2.17±0.23	1.18	3	0.78		1
	Emamectin+DEF	3.15 (2.60–3.94)	1.92±0.21	2.44	3	0.49		1
	Fipronil+PBO	2.11 (1.71–2.73)	1.91±0.23	1.91	3	0.59		1
	Fipronil+DEF	1.85 (1.48–2.45)	1.66±0.21	1.71	3	0.64		1
Field	Bifenthrin	127.62 (104.75–157.69)	1.74±0.17	1.74	4	0.78	11.72	
	Cypermethrin	317.45 (250.39–418.56)	1.39±0.14	1.73	5	0.88	64.79	
	Deltamethrin	209.12 (170.00–262.11)	1.55±0.12	7.93	6	0.24	15.87	
	Chlorpyrifos	16.31 (13.17–21.29)	1.81±0.22	0.02	3	0.99	8.82	
	Profenofos	39.85 (32.89–49.41)	1.84±0.16	7.74	5	0.17	22.02	
	Emamectin benzoate	124.59 (97.82–168.87)	1.54±0.17	0.38	4	0.98	43.11	
	Fipronil	43.24 (30.34–67.40)	2.01±0.19	7.56	4	0.11	22.29	
	Bifenthrin+PBO	21.94 (17.81–27.04)	1.65±0.16	0.56	4	0.97	3.33	6
	Bifenthrin+DEF	64.35 (41.91–100.77)	2.44±0.24	6.73	3	0.08	5.44	2
	Cypermethrin+PBO	43.86 (35.62–54.65)	1.53±0.13	8.02	5	0.16	11.42	7
	Cypermethrin+DEF	162.50 (124.05–233.37)	1.51±0.18	2.88	4	0.58	29.93	2
	Deltamethrin+PBO	38.76 (32.24–46.83)	1.82±0.15	5.79	5	0.34	4.90	5
	Deltamethrin+DEF	100.09 (80.84–128.62)	1.68±0.17	0.97	4	0.91	6.20	2
	Chlorpyrifos+PBO	6.19 (5.70–7.78)	1.85±0.21	1.60	3	0.66	2.44	3
	Chlorpyrifos+DEF	3.66 (3.03–4.41)	2.03±0.22	3.38	3	0.34	2.38	4
	Profenofos+PBO	14.77 (11.75–19.55)	1.63±0.17	4.15	4	0.39	7.50	3
	Profenofos+DEF	5.48 (3.72–8.13)	1.78±0.16	7.21	4	0.13	5.43	7
	Emamectin+PBO	60.61 (47.72–78.54)	1.36±0.15	1.57	4	0.81	18.59	2
	Emamectin+DEF	94.04 (73.89–125.83)	1.42±0.16	0.50	4	0.97	29.85	1
	Fipronil+PBO	34.28 (27.88–43.00)	1.65±0.16	4.49	4	0.35	16.24	1
	Fipronil+DEF	38.45 (30.88–49.27)	1.55±0.15	4.48	4	0.44	20.78	1

* synergism ratio calculated as [LC_50_ of insecticide used alone ÷ LC_50_ of insecticide + PBO or DEF].

## Discussion

The present study was conducted for the purpose to evaluate the toxicities of pyrethroids alone and in mixture with non-pyrethroids against the insecticide resistant dairy population of house flies. The antagonistic or synergistic interactions among the tested insecticides depended on the type of insecticides used, ratios and strains. In the laboratory susceptible strain, the combination between one pyrethroid and the other compounds produced an antagonistic interaction (in the ratio 1∶1), except the following combinations: bifenthrin/fipronil and cypermethrin/emamectin where the interactions were additive and synergistic, respectively. In contrast, all the mixtures of pyrethroid/non-pyrethroid produced a synergistic effect when used in the ratio of LC_50_: LC_50_, except the combination cypermethrin/profenofos. In the field strain, all the mixtures of pyrethroid/non-pyrethroid when used in the ratio of 1∶1, except the following combinations where responses were synergistic: cypermethrin/profenofos or emamectin or fipronil, and deltamethrin/emamectin. However, at the LC_50_: LC_50_ ratio, all the mixtures of pyrethroid/non-pyrethroid produced synergistic effect, except the combination bifenthrin/fipronil. The synergistic interaction between pyrethroids and organophosphate insecticides has previously been reported in different pests like *Bemisia tabaci* [22], *Culex quinquefasciatus* [4], *Helicoverpa armigera* [23], [24], *Musca domestica* [7], *Pectinophora gossipiella* [25], *Plutella xylostella* [26], *Spodoptera litura* [12], *S. littoralis* [27] and *Tetranychus urticae* [28]. However, the antagonistic interactions between pyrethroid and organophosphate have also previously been reported in different insect species like *B. tabaci* from Pakistan [29], *H. armigera* from Africa [6] and *S. littoralis* from Egypt [30]. The results of the present study also revealed that the toxicity of pyrethroids could be enhanced by the addition of new insecticides like emamectin benzoate and fipronil. Previously, Attique et al. [26] has documented the enhanced toxicity of bifenthrin and chlorpyrifos by new chemical insecticides in *P. xylostella*.

According to the results of present study the toxicity of bifenthrin, cypermethrin and deltamethrin against house flies could be enhanced by the addition of chlorpyrifos, profenofos, and in some cases by emamectin or fipronil, which reveal that these insecticides might be countering the resistance mechanisms in the present population. Corbett [31] proposed a general theory to explain the synergistic interactions among insecticides. According to this, one product or toxicant in the mixture interferes with the metabolic detoxification of the other toxicant, thereby synergizing the toxicity of the latter toxicant. In addition, insecticides from pyrethroid and organophosphate classes may be potential or competitive substrates for the same oxidase, as demonstrated by Kulkarni and Hodgson [32], thus potentiating the toxicity of the insecticide mixture. Moreover, it was also proposed that when pyrethroid/organophosphate mixtures applied against resistant insects, OP insecticide might bind to the monooxygenase that the first result in the activation of molecules, and then prevent the binding and subsequent degradation of pyrethroid insecticide by monooxygenase enzymes. These enzymes when bind to the OP insecticide could also lead to non-toxic metabolites by the process of hydroxylation of either oxon or thioate forms, which ultimately results in the degradation via oxidative ester cleavage. In this way, the binding of monooxygenase enzymes with OP insecticide would prevent or delay the degradation, and enhanced the toxicity of pyrethroid insecticide by competitive substrate inhibition mechanism [26], [32]. Previously it has been assumed that organophosphates, when used in combination with pyrethroids, inhibit the enzymes (monooxygenases and/or esterases) responsible for metabolic detoxification in different insect pests [6], [9], [10]. In the present study, the organophosphates largely overcame the resistance of pyrethroids, may be due to the inhibition of metabolic detoxification enzymes. We have no direct evidence of enzyme inhibition by organophosphates, but this hypothesis could be checked in the future studies.

The use of two enzyme inhibitors in the present study viz., PBO and DEF, against the field population of house flies largely overcame the resistance to the pyrethroids and organophosphates, which indicate that the resistance was mixed function oxidase or esterase based. However, both inhibitors did not synergise any insecticide against the laboratory strain. Previously, the effect of enzyme inhibitors in enhancing insecticide toxicities has been studied in different insect pests. In house flies, for example, the mixture of PBO with different pyrethroids increased the toxicity of cypermethrin, deltamethrin and permethrin [33]. Although PBO is a very good synergist, it should be used cautiously in field due to possible ill effects on non-target organisms. Moreover, the environmental protection agency (EPA) has categorized it as class C human carcinogen [34]. The occurrence of mixed function oxidase or esterase based resistance has been reported in different insect pests. PBO and DEF have also showed good synergism with cypermethrin in *Agrotis ipsilon* and *H. zea* [35], *S. litura* [12], [36] and *S. exigua* [37]. Compared with other insecticides, emamectin and fipronil seem to resist the enzymatic attack, as there was no synergism by PBO and DEF for fipronil, and DEF for emamectin and fipronil. Such type of results indicates that other mechanisms of insecticide resistance like decreased cuticular penetration and target-site insensitivity may be of major importance in the present population for emamectin and fipronil; however, further studies are required to confirm the exact mechanisms of resistance to these insecticides in Pakistani populations of house flies.

The findings of the present study showed the presence of resistance to all the insecticides under investigation that suggest that multiple resistance mechanisms may be present in field population of house flies. In conclusion, the occurrence of resistance to different insecticides in disease vectors of public health importance has stressed the need to search out ways and develop strategies to manage resistance in field conditions [2]. The results reveal that the toxicity of bifenthrin, cypermethrin and deltamethrin to house flies could be enhanced by the addition of chlorpyrifos, profenofos, and in some cases by emamectin or fipronil. Insecticide mixtures could be very helpful in the management of house flies, especially in cases where synergistic interactions may occur between insecticides. Since, owing lack of systematic management plans for house flies in Pakistan [16], [17], such resistance management tools are not in practice here. Recently, it has been reported that house flies got indirect exposure to chemicals used for the management of different dairy pests [16]. Insecticide mixtures, as a resistance management tool, should be given importance whenever a systematic management plan for dairy pests including flies will be designed by the policy makers. However, the fact of the occurrence of multiple resistance when insecticide mixtures are used for long, and effects on beneficial organisms should not be ignored [26]. For this, alternative tactics for the management of insecticide resistance like fine scale mosaics and/or rotation of insecticides should also be given importance. Moreover, studies on basic and operational aspects of interaction between insecticides in mixtures should be strengthened to set up adequate resistance management plans under field conditions.
