# Correction: Insecticide Mixtures Could Enhance the Toxicity of Insecticides in a Resistant Dairy Population of *Musca domestica* L.

**DOI:** 10.1371/annotation/14f1545c-cbe3-4783-b7c6-f6f9e4c4a1f4

**Published:** 2013-08-22

**Authors:** Hafiz Azhar Ali Khan, Waseem Akram, Sarfraz Ali Shad, Jong-Jin Lee

The correct version of the title is: Insecticide Mixtures Could Enhance the Toxicity of Insecticides in a Resistant Dairy Population of *Musca domestica* L.

The first author Hafiz Azhar Ali Khan should also be affiliated with the institution: Institute of Agricultural Sciences, University of the Punjab, Lahore, Pakistan 

